# Sentinel-1A for monitoring land subsidence of coastal city of Pakistan using Persistent Scatterers In-SAR technique

**DOI:** 10.1038/s41598-022-09359-7

**Published:** 2022-03-28

**Authors:** Muhammad Afaq Hussain, Zhanlong Chen, Muhammad Shoaib, Safeer Ullah Shah, Junaid Khan, Zheng Ying

**Affiliations:** 1 School of Geography and Information Engineering, China University of Geosciences (Wuhan), Wuhan, 430074 China; 2 State Key Laboratory of Hydraulic Engineering Simulation and Safety, School of Civil Engineering, Tianjin University, Tianjin, China; 3 Ministry of Climate Change, Government of Pakistan, Islamabad, Pakistan; 4 School of Engineering, China University of Geosciences (Wuhan), Wuhan, 430074 China

**Keywords:** Environmental sciences, Hydrology, Natural hazards

## Abstract

Karachi is located in the southern part of Pakistan along the Arabian Sea coast. Relevant institutions are concerned about the possibility of ground subsidence in the city, contributing to the comparative sea-level rise. So yet, no direct measurement of the subsidence rate and its relation to city submergence danger has been made. SAR (Synthetic Aperture Radar) interferometry is a powerful method for obtaining millimeter-accurate surface displacement measurements. The Sentinel-1 satellite data provide extensive geographical coverage, regular acquisitions, and open access. This research used the persistent scatterer interferometry synthetic aperture radar (PS-InSAR) technology with Sentinel-1 SAR images to monitor ground subsidence in Karachi, Pakistan. The SARPROZ software was used to analyze a series of Sentinel-1A images taken from November 2019 to December 2020 along ascending and descending orbit paths to assess land subsidence in Karachi. The cumulative deformation in Line of Sight (LOS) ranged from − 68.91 to 76.06 mm/year, whereas the vertical deformation in LOS ranged from − 67.66 to 74.68 mm/year. The data reveal a considerable rise in subsidence from 2019 to 2020. The general pattern of subsidence indicated very high values in the city center, whereas locations outside the city center saw minimal subsidence. Overall, the proposed technique effectively maps, identifies, and monitors land areas susceptible to subsidence. This will allow for more efficient planning, construction of surface infrastructure, and control of subsidence-induced risks.

## Introduction

Analyzing and monitoring subsidence in large cities allows meaningful insights for mitigating the probable loss of property and life due to increasing development rates in many regions around the globe^1^. A range of variables that could produce urban subsidence includes human and natural reasons such as building loads^2–4^, lithology^2,5–7^, groundwater consumption^3,8,9^, tectonic activity^3,10^, dewatering^11^, seasonal effects^12^, mining activities^13^. Each of these variables might have a unique impact on surface deformation. Worldwide, migration of people from villages to cities regions in search of better employment access and opportunities to the standard of living amenities. Natural resources are being harmed due to unplanned and unregulated expansion in an area. The need for daily water extraction is rising, and consequently, excessive groundwater extraction is producing surface liquefaction^14–17^.

Land subsidence (LS) associated with variables such as seismic activity and groundwater extraction may influence human life, the environment, and urban infrastructure. The Indo-Gangetic plains are prone to subsidence^18^. The main reason behind this is high population density leading to over-extraction of groundwater. Numerous modern researchers have investigated the volume of global groundwater extraction in order to investigate the role of reduced continental water storage to sea-level rise^19–21^. Groundwater extraction increases the expense of pumping or causes wells to dry up, influencing users^22^; reduces groundwater flow to streams, wetlands, and springs, harming ecosystems; and causes land subsidence^23^, diminishing storage irrevocably and possibly destroying infrastructures^24^. However, the extent of the harm is not usually understood until it is too late. Pakistan is not immune to Land Subsidence^25–27^. However, no complete information on the features of LS is currently accessible and valuable in mitigation efforts and planning, which is primarily hampered by the lack of accurate chronological data. The land subsidence in coastal regions can accelerate the pace of relative Sea Level Rise (SLR) and increase the frequency and severity of coastal floods^28^. Since the previous decade, studies have raised concerns regarding flooding, coastal land subsidence, erosion caused by coastal development, and natural risks such as SLR^29^. Due to a lack of awareness about the severity of the problem and a scarcity of up-to-date information regarding human-induced LS, no scientific research has been conducted to measure the LS rate. Because the variables that cause LS vary by area, this model was developed to adapt subsidence inputs based on the specific features of a studied region. The reason for subsidence and the interaction, connections, and integration between these causative variables must be thoroughly investigated to achieve this aim.

In the last two decades, Remote Sensing (RS) technology such as synthetic aperture radar interferometry (In-SAR) has proved their significant potential in various fields, including but are not constrained to studying groundwater extraction^30^, deltaic LS^31,32^ and landslide deformation^33,34^. Because of its capacity to acquire data regularly and repeatedly, it makes historical data archives available. Several sophisticated approaches, such as Small Baselines Subset Approach^35^, Parallel Small BAseline Subset (P‐SBAS)^36^, Permanent Scatterers-InSAR^37^, Spatio-Temporal Unwrapping Network^38^, and the Interferometric Point Target Analysis^39^, have now been developed.

Differential Interferometry Synthetic Aperture Radar (D-InSAR) is a method that uses space to collect large-scale surface micro-deformation information^40^. The D-InSAR approach results from quantitative advancements in RS, particularly microwave RS. However, as research has progressed, various drawbacks of classic D-InSAR have gradually been revealed, limiting its applications. Such as atmospheric interference, spatial decorrelation, and temporal decorrelation^41,42^. To address these issues, researchers suggested PS-InSAR^41^. As dozens of acquisitions may be utilized to produce multiple pairs, this technique eliminates the impact of decorrelation and atmospheric effects in standard D-InSAR. It assures that the approach may be employed even when the critical baseline is exceeded by the baselines of numerous pairs of acquisitions. Compared to GPS and leveling technologies, PS-InSAR can get a ten-year sequence of surface deformation data from hundreds of scenes. PS point density is significantly higher than data point density produced by GPS and standard leveling measuring techniques. This technique not only saves money, but also ensures sufficient precision for ground deformation monitoring, as confirmed by GPS and leveling technology^42^. As a result, in recent decades, PS-InSAR has been extensively used in a variety of applications, including bridge detection, building deformation monitoring, and surface subsidence^43–45^.

Many cities have used InSAR to monitor LS lead by human proceedings such as groundwater extraction and development^8,46–50^. The generation of time-series of subsidence observation with high temporal precision primarily depends on sophisticated multi-temporal interferometry approaches, including persistent scatter interferometry (PSI)^51–53^. PSI has been shown to accurately characterize linear deformation (very slow-moving or slow) in locations with a high density of “permanent scatters” (e.g., buildings)^8,54,55^. Combining these two approaches is preferred for characterizing the complicated deformation process over large-scale land development areas and fast urbanization when radar targets are neither steady nor restricted. Precise ground velocity calculations can help to minimize ambiguity in liquefaction models. Microwave remote sensing data allows for accurate measurement of surface movements. To identify subsidence in the research region, our model relies on the PS-InSAR technique^37^. PS-InSAR is a SAR-based deformation observation approach that identifies and exploits stable targets as persistent scatterers in a temporal sequence of interferograms to solve the temporal and geometrical decorrelation issues of differential interferometric SAR (D-InSAR). This method can detect surface deformation over a large region with millimeter-level accuracy^56^.

Pakistan’s coastline is densely inhabited^57^, and land deformation has been recorded in recent years^58,59^. Karachi, Pakistan’s largest metropolitan region, suffers from unsustainable and unregulated urban growth. Some of its beachfront lands have recently been recovered for tourist spots, commercial, and residential—coastal erosion, waterlogging and uncontrolled land reclamation increase the likelihood of LS in Karachi^57,60,61^. Consequently, measuring and monitoring LS in a dense urban coastal metropolis (Karachi) is critical for sustainable urban expansion. As a result, this work aims to estimate land deformation in Karachi on a multi-temporal scale to identify regions sensitive to land deformation.

Ground subsidence is a significant problem in several Pakistani cities, including Karachi^62^, with about 16.5 million (Census report 2017), and is home to many educational institutions, including military, medical and engineering colleges. The local administration has developed various water supply projects collaborating with the Japan International Cooperation Agency (JIPC 2008) to support urban expansion^62^. Some investigators have monitored LS rates in Karachi city using InSAR techniques during the last decade. Nevertheless, most of those studies relied on incorrect data, introducing uncertainty into the resulting subsidence rates. These studies used an insufficient number of ascending data sets and Envisat data set. In this regard, in the recent study compared to the earlier studies on Karachi city, we: (1) used PSI technique and concentrated primarily on urban areas, (2) used both descending and ascending track, (3) used Sentinel-1 SAR data, (4) estimated the subsidence rate in current years, and (5) computed LOS and vertical deformation.

This research was carried out to monitor ground subsidence in Karachi, Southern Pakistan, from 23 November 2019 to 23 December 2020 and 20 December 2019–26 December 2020, with both ascending and descending tracks. Because the satellite can see the same target area from different positions, with incidence angles ranging from vertical (~ 23°) to horizontal (~ 45°)^63^ in the East–West direction, descending and ascending track images are used to improve visualization and truly comprehend deformation from different directions^64^. In addition, our work assesses the capacity of applying the PS-InSAR technology for LS investigations along coastal regions^65–67^.

## Study area

**Figure 1:**
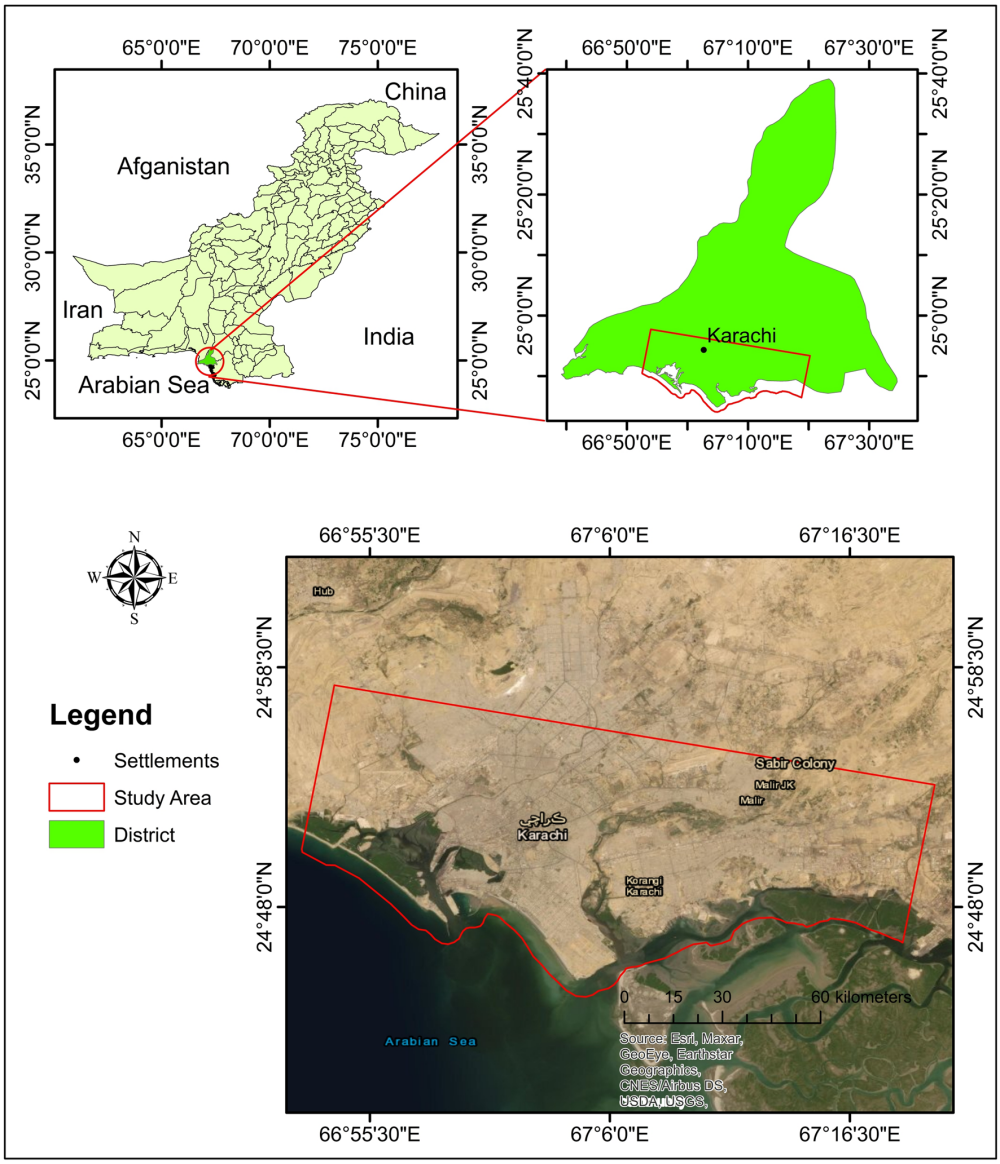
The geographical location of the study area.

**Figure 2:**
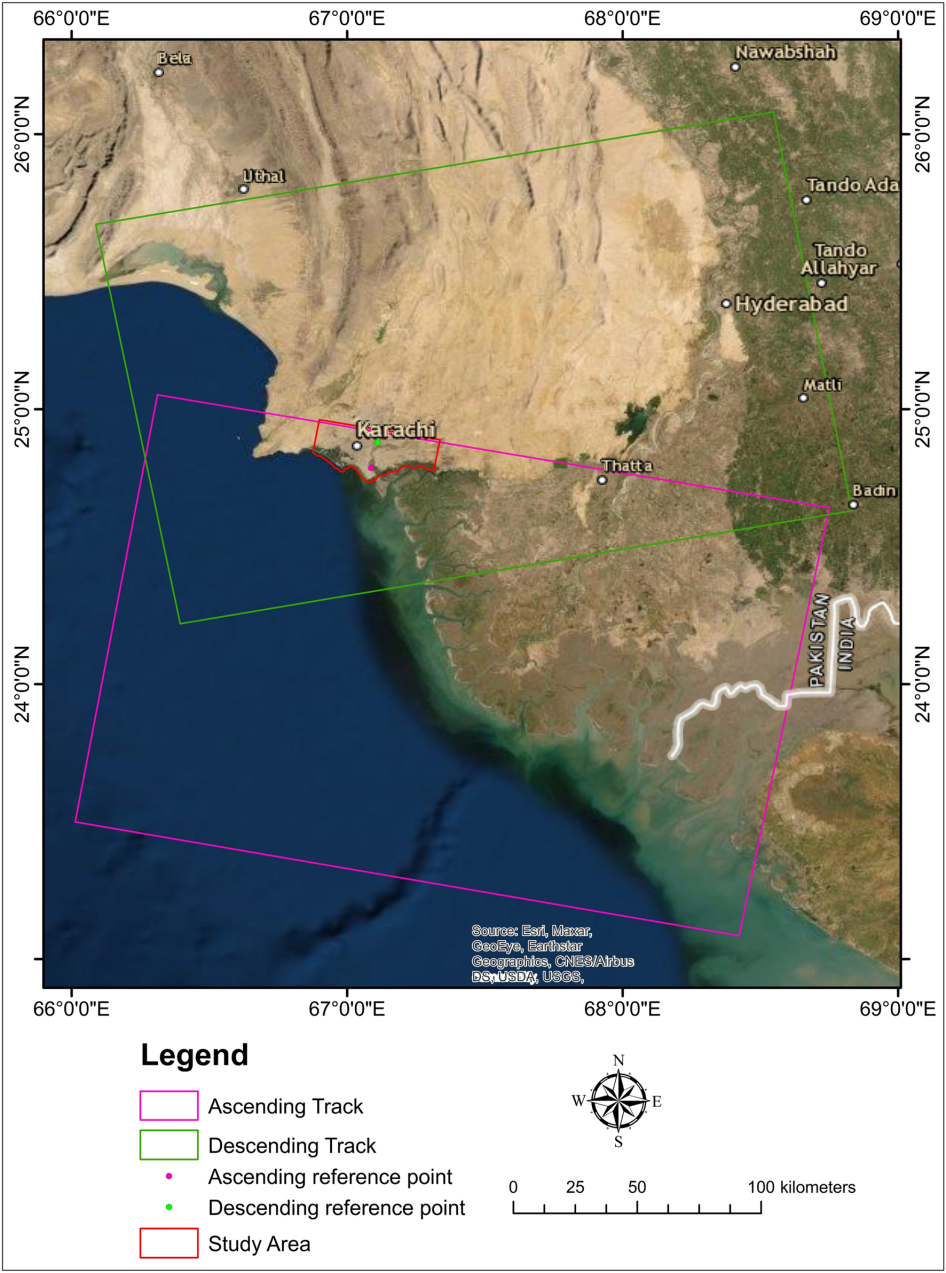
Research area (red color) with a footprint of the master area and reference points.

Geographically, the extent of the study area is marked by latitudes from 24° 43′ N to 24° 57′ N and longitudes from 66° 53′ E to 67° 20′ E (Figs. 1 and 2), covering an area of about 737 km^2^. Karachi is an industrial hub of Pakistan with a population of 16.05 million. It is the largest urban city of Pakistan, divided into six districts (Table 1). It is located approximately 128 km from the mouth of river Indus while its southern and south-western part is along the Arabian Sea. The topography is flat as slopes range between 0° and 4° except for hilly areas in the northwestern parts where slopes range up to 64°. The length of the coastline from Karachi Port Trust (KPT) to Korangi is ~ 25 km.

**Table 1:** Overview of the district-wise number of Households (hh), Persons per house, and population in Karachi (Census, 2017).

Districts	No of hh	Person per house	Population
Karachi south district	327,518	5.47	1,791,751
Malir district	338,257	5.94	2,008,901
Korangi district	421,618	5.83	2,457,019
Karachi east district	509,647	5.71	2,909,921
Karachi central district	539,127	5.51	2,972,639
Karachi west district	634,459	6.17	3,914,757
Total	2,770,626	5.80	16,054,988

The research area is located in the world’s northern tropical zone. This tropical zone encompasses most of the world’s desert belts, with significant mountain belts oriented north–south on the west. The study region has a moderate climate, little precipitation, and extremely scorching summers. However, due to their proximity to the sea, these locations maintain a high degree of humidity. Based on the previous 50 years of data, the average annual rainfall in Karachi is around 200 mm, and winter temperatures range between 24 and 28 °C, while summer temperature ranges between 34 and 38 °C (Pakistan Metrological Department)^68^.

### Geological setting of the area

**Figure 3:**
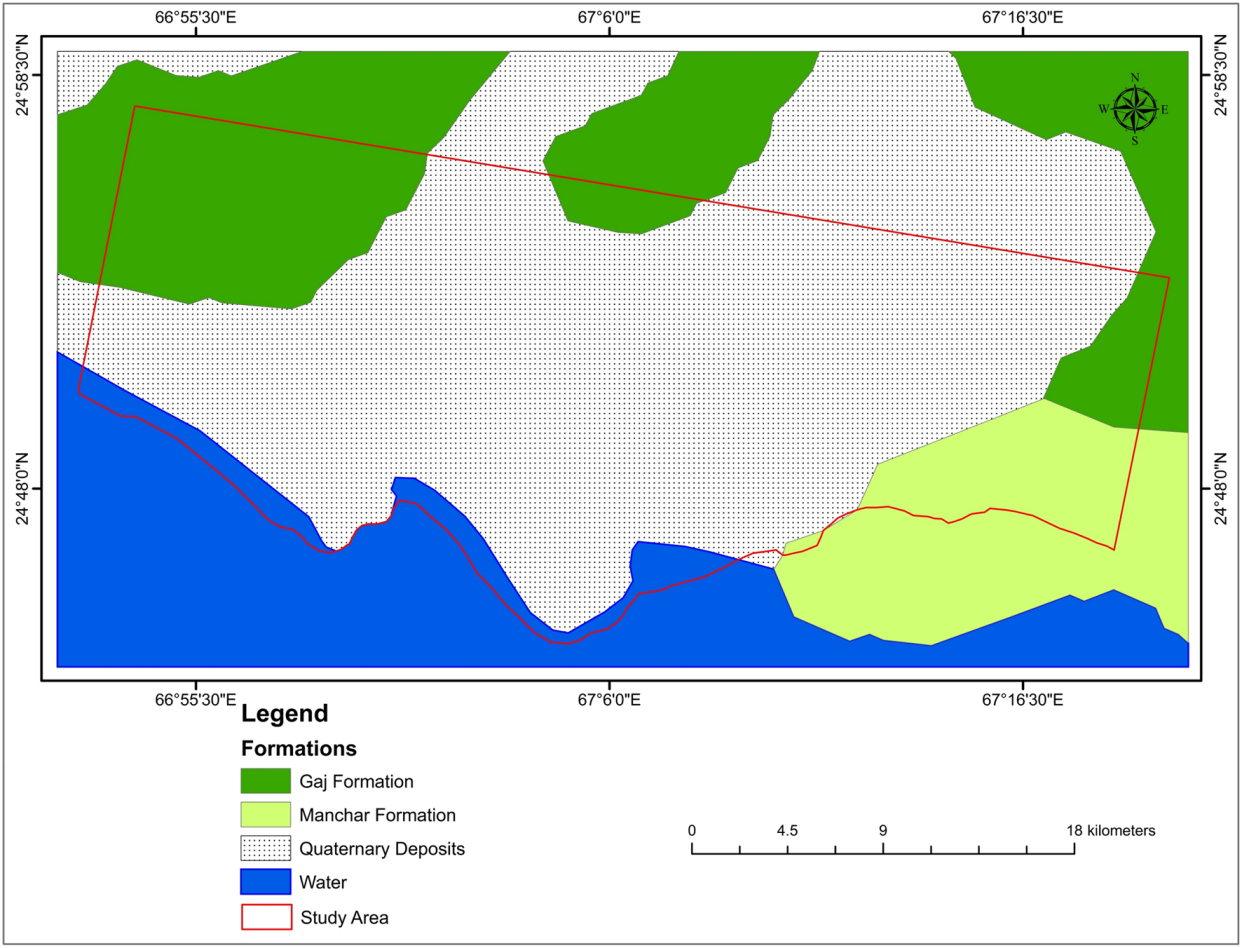
Geological map of the research area.

Karachi city lies on the southern side of the Indus basin. The research area is mainly composed of two formations: the Gaj Formation (Miocene age), the Manchar Formation (Plio-Pleistocene age), and the Quaternary deposits (Fig. 3). According to the Geological Survey of Pakistan, the research area is composed of the following lithology.

### Gaj formation

Gaj formation Composed of gravel, Sandstone, Limestone, and Clay. The gravels are deposited over various degraded bedrocks, such as shales, sandstones, and limestones. Areas of silty or worn gravely soil cover the inter-mound patches. The gravel deposits appear to be the lateral planted depositional surface formed across the eroded bedrocks by a free-flowing and ever-shifting river system. There is more than one level of these gravels, which might be the terrace remains created by the consecutive change of base level of erosion. Limestone in Gaj formation is light brown to a golden brown, and sandstone is grey and brown. The color of Clay is grey-brown and yellow in Gaj formation.

### Manchar formation

Manchar Formation comprises sand, sandstone, silt, conglomerates, and gravel patches. The surface has formed on the eroded margins of crumbly sandstone and shales from the Plio-Pleistocene Manchar formation. The warping in the Manchar Formation is relatively mild. Pleistocene conglomerates exist unconformably on the Manchar Formation eruptive surface. The color of sandstone in the Manchar Formation is grey and greenish-grey.

### Quaternary sediments

Quaternary sediments or Recent Alluvium are mainly composed of sand, silty sand, sandy silt, deltaic, coastal, and eruptive mud deposits of recent age with minor clay components, most likely as a result of coastal geographic control and domination of aeolian deposits from the shore^69^. It is tectonically stable. It creates the platform cover in the Indus basin, and the valley fills in the intermountain basin.

## Methodology

### Data processing

In this investigation, we employed Sentinel-1 C-band SAR pictures recorded along both ascending and descending orbit tracks (Alaska Satellite Facility: https://asf.alaska.edu/about-asf/). To complete the analysis in C-band data, the PSI^70^ requires at least 20 SAR pictures^71^. The PSI monitors surface subsidence over months or years, accounting for signal noise, atmospheric, and topographic impacts. This sensor has a ground resolution of around 20 m in the azimuth direction and 5 m in the range direction^72,73^. This sensor has several acquisition modes, including interferometric wide (IW), wave (Wave), extra-wide swath (EW), and strip map (SM). When comparing the IW mode to other acquisition modes, it was discovered that the IW mode requires more data processing for co-registration of images with a high precision of up to 0.001 pixels^74^. The 34 images from the ascending track (23 November 2019–23 December 2020) and 32 images from the descending track (20 December 2019–26 December 2020) were collected for this study.

**Figure 4:**
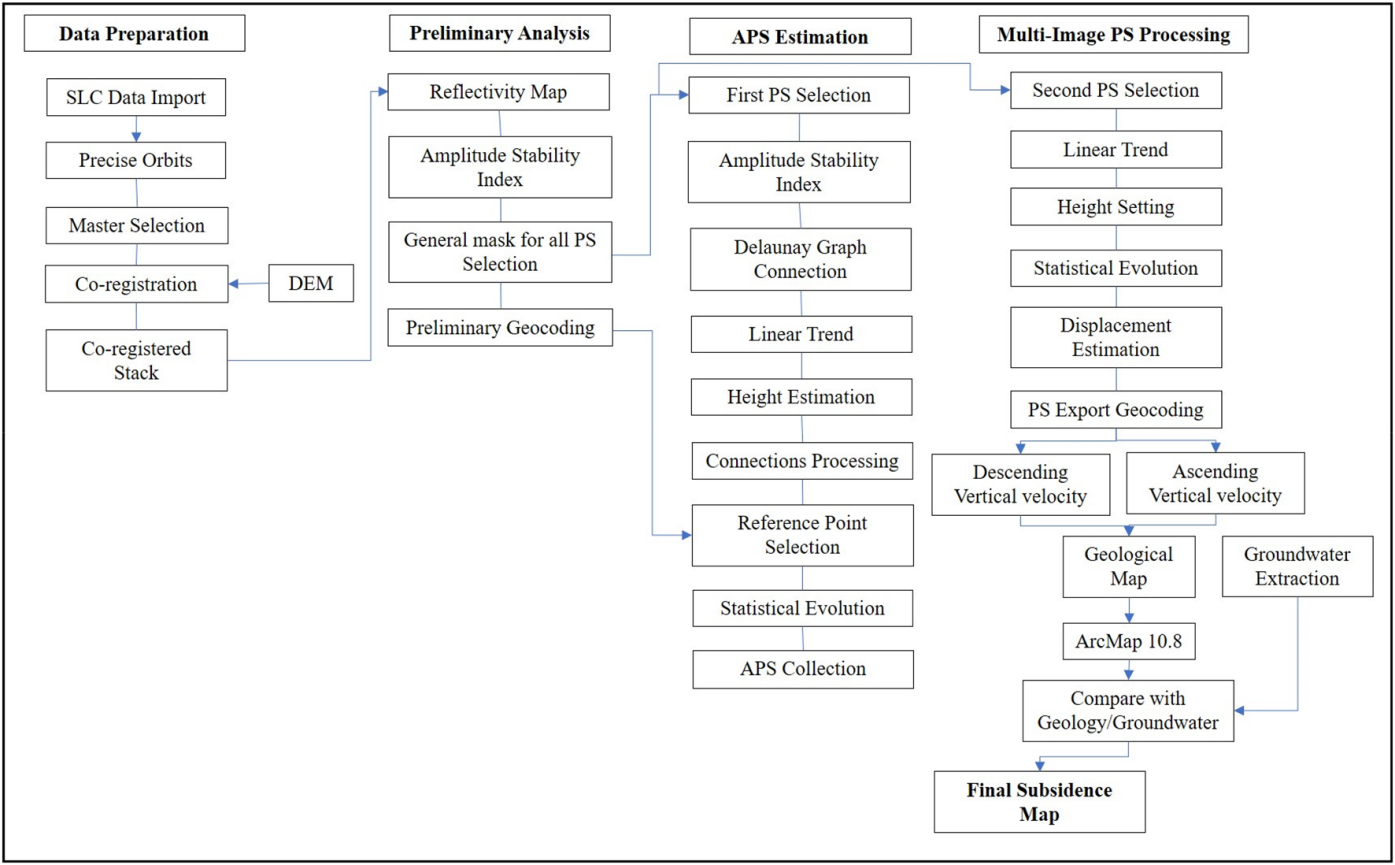
Flowchart of the research.

The IW acquisition mode was used to acquire all of the images. Sentinel-1, IW mode covers a single scene with a coverage area of 250 km^2^. The Terrain Observation by Progressive Scan (TOPS) mode divides the single scene into three sub-swaths. Because SAR imagery has a high temporal and spatial resolution and a short returning time, it may be used to study subsidence events from satellites^75^. SARPROZ software (https://www.sarproz.com/sarproz-faq) was utilized for this research, which is highly beneficial for InSAR data studies and commercial software^74^. SARPROZ has been successfully used, for example, by Qin et al.^76^ to create a liquefication map of Hong Kong demonstrating the PSI’s precision to the millimeter level. It employs the concepts of the PS-InSAR method as detailed by^41,77^. The PS-InSAR process involved the preparation of data, data analysis, APS Estimation, and Multi-image Processing. The methodology followed in the research is shown in Fig. 4.

**Figure 5:**
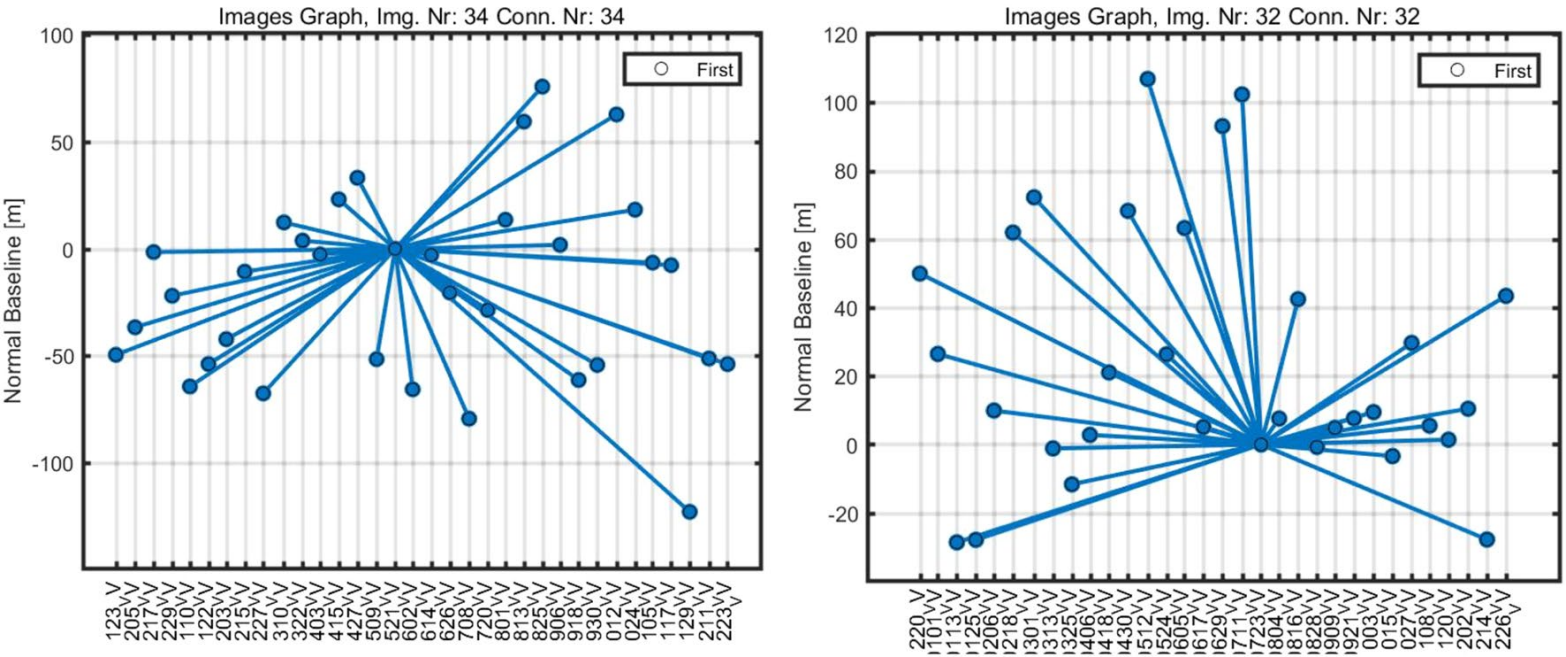
Star graphs depict the perpendicular/temporal baseline distribution of data pairs.

Importing SLC data with accurate orbits is one of the processing steps in data preparation. Imagery with the same rotations, both descending and ascending, was chosen for this study. However, both descending and ascending images cannot be analyzed simultaneously. Following that, the polarization of images was determined based on orbit information, and master and slave images were chosen. The master images covering the study region were retrieved first, followed by slave images covering the same common area as the master image. In this case, a star graph was generated between the slave image and the master image (Fig. 5). A specified region was evaluated and co-registered during the co-registration phase^74^.

Atmospheric phase screen (APS), orbital inaccuracies, and other factors were calculated and removed. Following that, the phase stability was evaluated. Absolute amplitude levels were primarily indifferent to generating processing disruptions^78^. As a result, it was predicted that the pixels would have identical amplitudes and reduced phase dispersions for all of these acquisitions. PS is chosen in the SARPROZ software process based on the amplitude stability index (ASI). Various atmospheric phase delays impact SAR pictures during acquisitions, and signal interruptions, such as radar signals being affected by aerosol particles, often happen^74^. Multiple spatial–temporal filtrations are employed to compute the Atmospheric phase screen to prevent these disruptions in dataset^79^. At this point, Atmospheric phase screen findings are discarded, whereas linear deformation velocities and topographic height effects are calculated from the advanced stages^74^.

**Figure 6:**
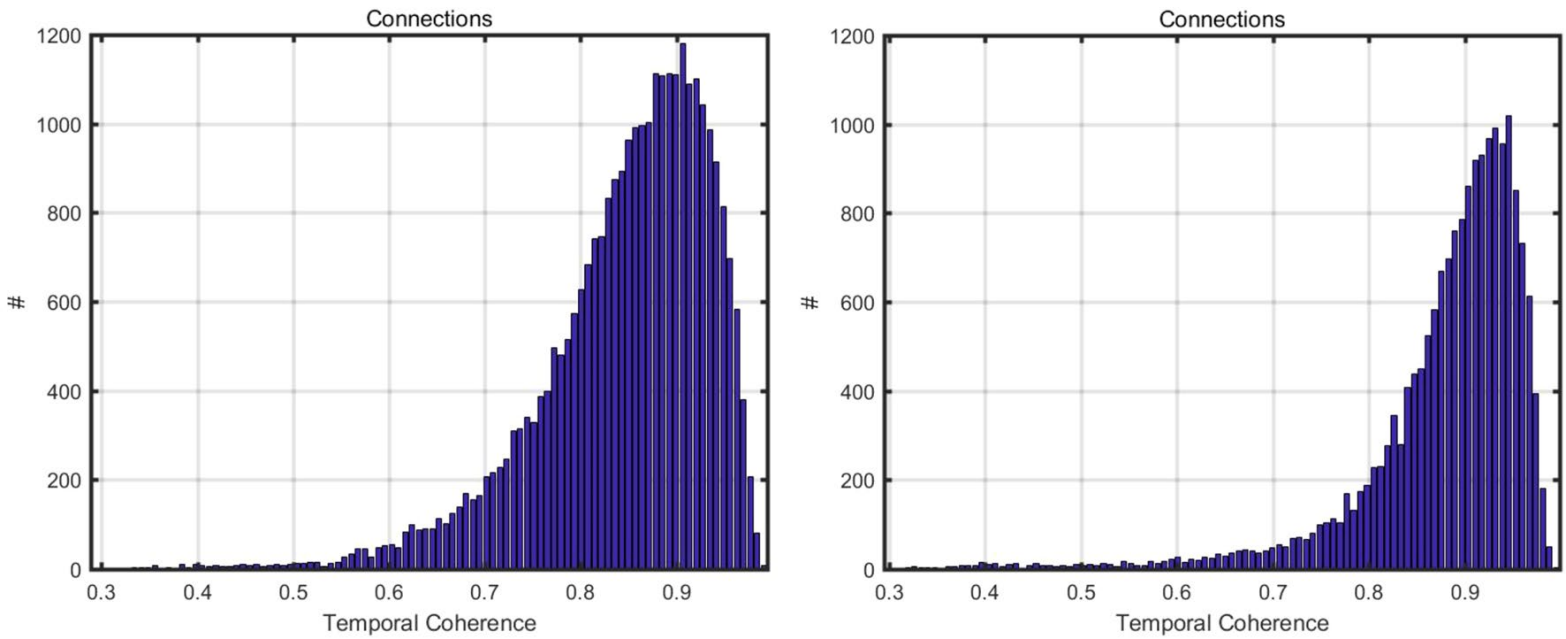
The graphs between the temporal coherence and connections for Ascending path on the left side and Descending path data on the right side.

For this objective, an acceptable threshold, ASI > 0.75, is proposed as a reference for selecting the initial PSs^37^. In our research, ASI > 0.6 was used to choose PSs. This restrictive parameter estimation fulfills by allowing just a limited number of PS points, which is essential for calculating the proper APS. Following the selection of the first PS, it is necessary to build a reference network by connecting PSs using Delaunay triangulation at this point. This is continued by removing the calculated linear model (residual height and linear displacement velocities) and estimating APS from the phase residual using an inverse network. It is also critical to establish one point of reference and determine its velocity here. Following graph inverting and APS elimination, temporal coherence evaluation of PSs was performed to assess APS integrity, yielding an acceptable outcome with a coherence greater than 0.7 (Fig. 6).

The second-order PS points were chosen in the Multi-Image Sparse Point Processing stage. At this point, ASI > 0.6 criteria were used to obtain denser PS points^78^. To the removal of APS, the same parameters and reference points were utilized while processing the APS estimate. Ultimately, all PS points were geocoded and overlaid on Google Earth, and only PS points with coherence 0.70 or higher were chosen for the resulting subsidence map^78^.

Finally, the identified deformation regions were converted into an external reference framework, i.e., geographical coordinates. The resulting ground displacement map and geology map overlay and loaded into Geographic Information System (GIS) for further investigation. The GIS study included combining PS-InSAR findings with a geological and groundwater extraction^62^ to evaluate and confirm the observed subsidence regions, which were then assessed in the geological setting of Karachi. The results of the preceding stages were then integrated with various information layers in ArcGIS. These layers were utilized to evaluate the research region’s geological formations and Groundwater extraction and their connection with PS-InSAR-estimated subsidence.

## Results and discussion

We employed PS-InSAR, as explained above, implemented in SARPROZ for deformation monitoring in this region, which allowed us to discover deformation zones in Karachi City. The green color represents the stable spots determined by a stability threshold range (from − 10 to 10 mm/year). PS-InSAR uses a reference point to calculate and identify movements in the region; therefore, a stable point is chosen as a reference point to compare with the motions of other points in the area. While using this method, temporal coherence must be sufficient for further evaluation. PS points with temporal coherence > 0.7 were regarded as reliable, with a lower likelihood of mistake^74^.

**Figure 7:**
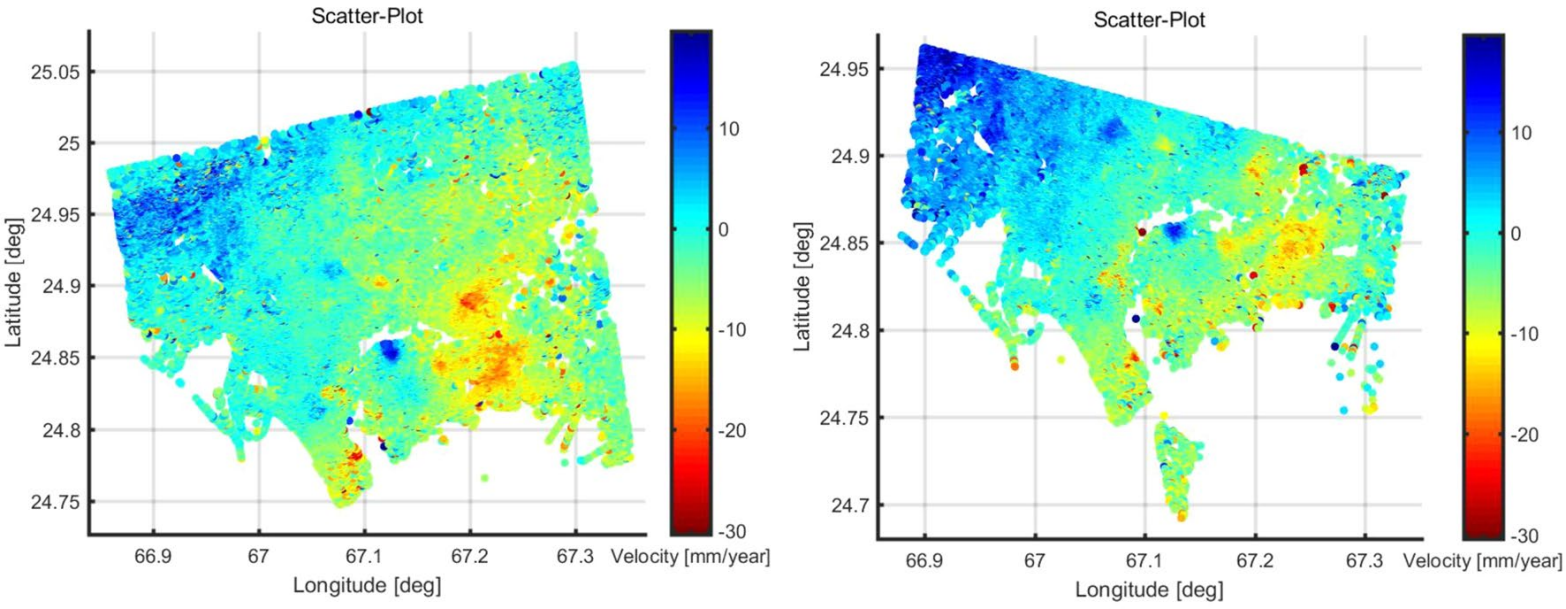
The scatter plots of Sentinel-1 data ascending and descending path on the lift to the right side (2019–2020).

When measuring motion along the LOS with PS points, it was discovered that the movement in the opposite direction of the sensor was negative, as shown in red. Other stable spots within the research region that reflect practically minimal movement are depicted in Fig. 6 (blue to light blue). In contrast, sites that showed comparably significant movement relative to the blue dots but lesser movement relative to the red dots are designated as yellow to dark yellow and orange. Subsidence in Karachi was measured to be between − 20 and − 30 mm/year (Fig. 7). The scatter plot data indicate that there was significant subsidence in Karachi City.

**Figure 8:**
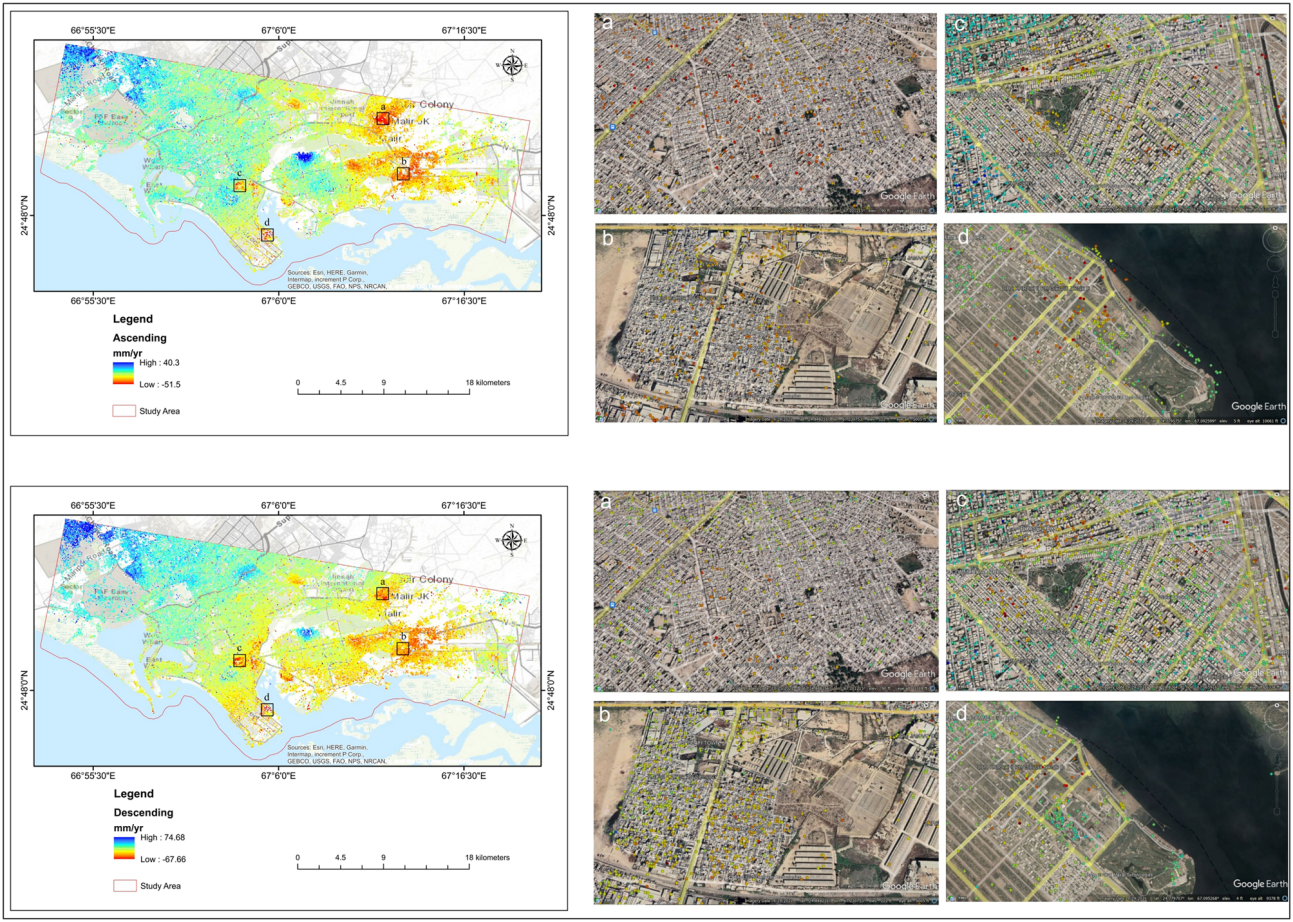
Land subsidence in the research area from the Ascending path (a, b, c, d) and Descending path (a, b, c, d).

During the analysis period, the subsidence map derived from both ascending (path number 42) and descending (path number 78) paths revealed a significant number of PS locations in the studied region (Fig. 8). The rectangular portion of scatter plots from ascending and descending is placed onto Google Earth in the research region. Figure 8 shows a thick points cloud in the research region; the findings in both the descending and ascending paths demonstrate that most of the area is stable (marked in blue), mainly upland. While the main habitation sites along the main road are shown in red, the comparatively high subsidence region is not. The color ramp represents movement and the relative stability of the PS points (red = high, blue = steady, yellow/light green = low).

**Figure 9:**
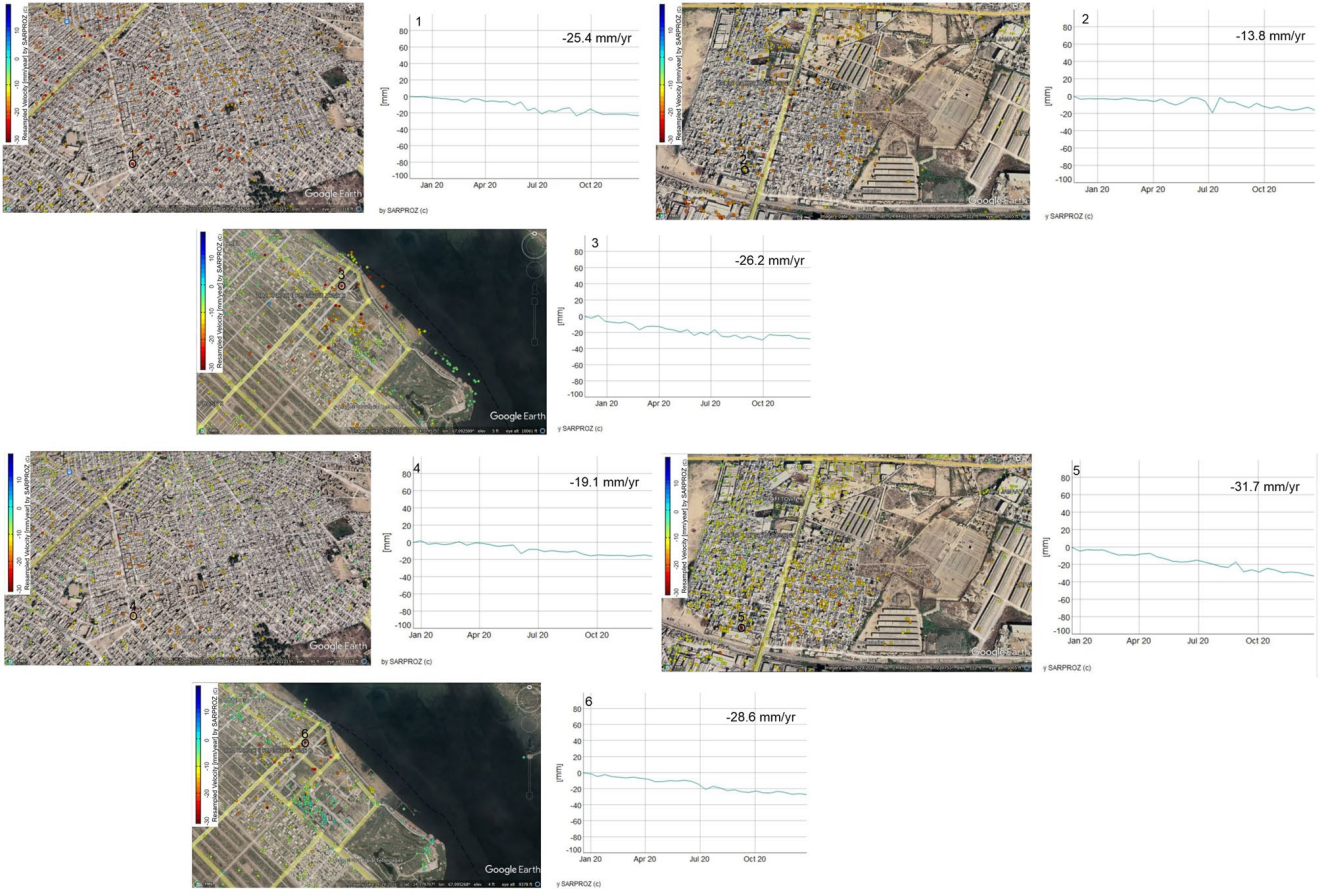
PS points are selected in Ascending path (P1, P2, P3) and descending path (P4, P5, P6).

Six PS locations (1, 2, 3, 4, 5, and 6) from the subsidence region were chosen from the descending and ascending findings in the research area (Fig. 9). The PS points, in this case, illustrate the relative movement and stability (red = high, blue = stable, yellow/light green = low) in comparison to the surrounding. Figure 9 depicts subsidence analyses along with these six PS locations.

Red dots show the subsidence area in Fig. 8. The acquired data illustrate differences in ground subsidence from point to point across the research periods. Figure 9 depicts the subsidence along with these sites, ascending points (1, 2, 3) and descending points (4, 5, 6). Points 1 and 4 are located in the northernmost part of the study area, where subsidence reached − 25.4 mm/year and − 19.4 mm/year, respectively. While points 2 and 5 are located in the central parts of the research area, where subsidence reached − 13.8 mm/year and − 31.7 mm/year, respectively, during the study analyzed period. PS sites 3 and 6 are located in the southern portion of the research region and had subsidence rates of − 26.2 mm/year and − 28.6 mm/year, accordingly, during the study period. The data show that subsidence was significantly higher in the city’s center, whereas it reduced in the northern and southern areas of the city.

The subsidence along these six PS locations was investigated, and the results demonstrate fluctuations in subsidence from 2019 to 2020. Figure 9 depicts the visual representations of these six points. The graphs clearly illustrate those points 2 and 5 have considerable subsidence and are positioned in the center of the research region, whereas points 1, 3, 4, and 6 have minor subsidence over the study period.

### Vertical velocity of Karachi

**Figure 10:**
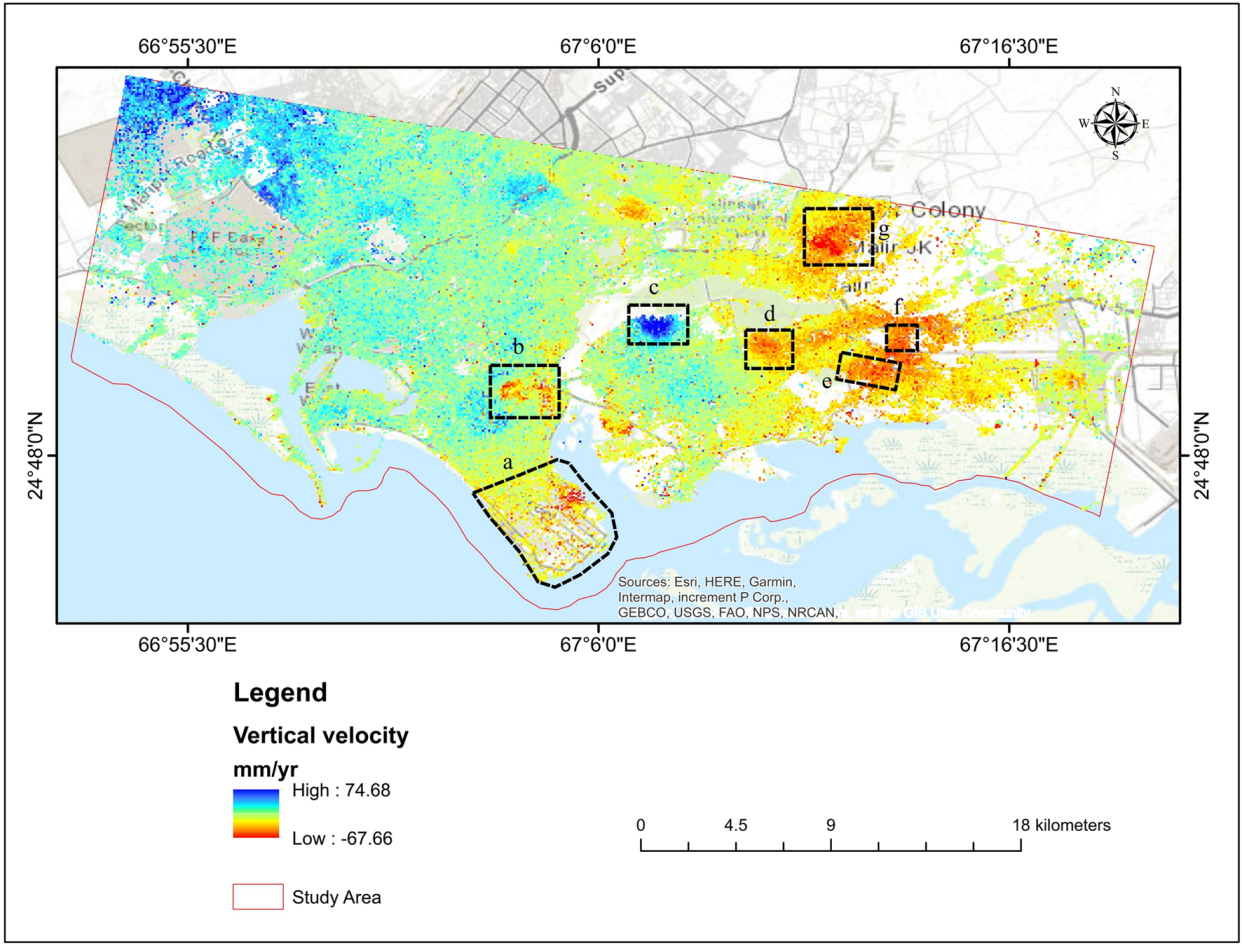
Vertical deformation (subsidence) in Karachi City presented on the google earth image.

Figure 10 depicts the vertical deformation map composed of descending and ascending Line of Sight (LOS) geometries; positive value shows steady to gentle uplifting movement, while a negative value shows liquefication/subsidence. The vertical deformation in LOS ranged from − 67.66 to 74.68 mm/year. The surface of Karachi is stable in the western part. The research area has the highest evaluated subsidence value of − 67.66 mm/year. The high subsidence areas are divided mainly Malir Colony, Radio Pakistan Colony, Sherpao Colony, Sector 7-A, and Sector 28, and parts of southern DHA Karachi Phase 4, and DHA Karachi Phase VIII Zone area. Subsidence in Karachi takes place mainly in the newly urbanized areas.

This research indicates that some variables influence and are accountable for ground subsidence in mega-city Karachi, Pakistan. Groundwater extraction to meet human demands, natural consolidation of quaternary alluvium, soil loss during wet seasons, infrastructure load, and unplanned building development are contributing causes.

**Figure 11:**
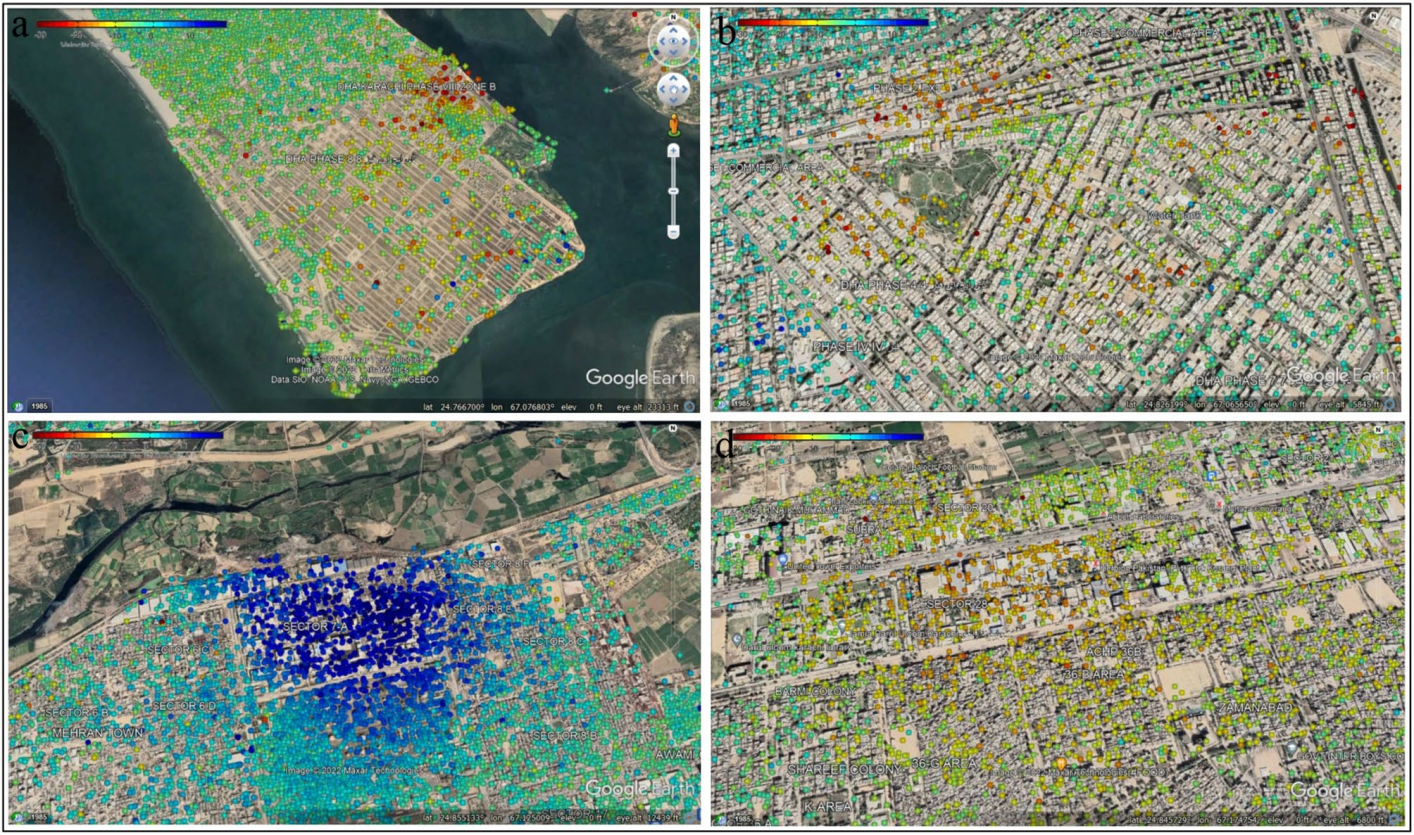
Vertical displacement in Karachi, (**a**) DHA Karachi Phase VIII Zone B, (**b**) DHA Karachi Phase 4, (**c**) Sector 7-A, (**d**) Sector 28.

**Figure 12:**
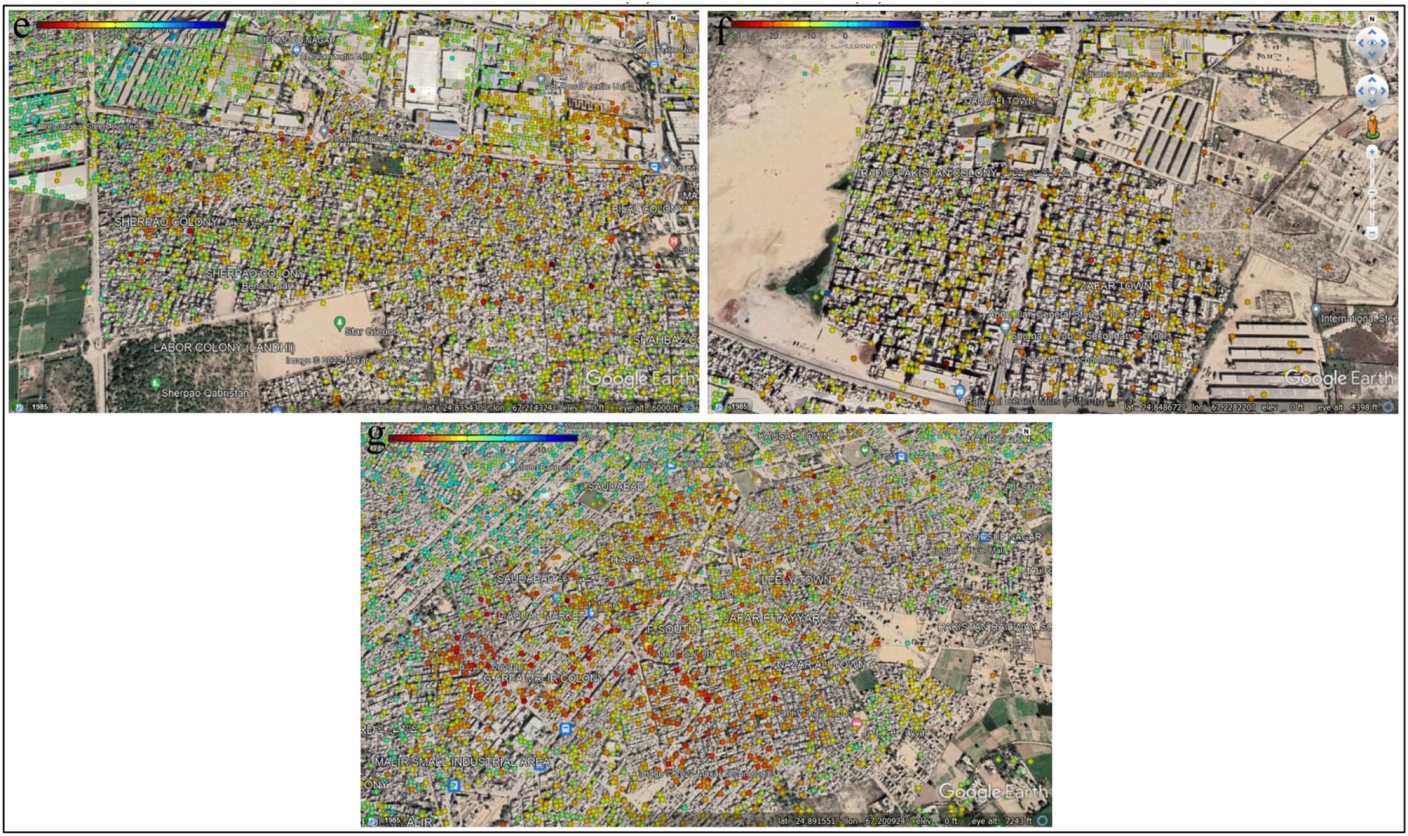
Vertical displacement in Karachi, (**e**) Sherpao Colony, (**f**) Radio Pakistan Colony, (**g**) Milar Areas.

The study area was divided into zones to understand better Karachi's land deformation (Figs. 11 and 12). The highest displacement observed is − 28.6 mm/year in DHA Karachi Phase VIII Zone B and − 27.7 mm/year in DHA Karachi Phase 4. In the nearby river Malir on the west, which is composed of gravel used for construction intents, the DHA Phase-VIII has bottom gravels whose concretion procedure is prolonged. As a result of the compaction and consolidation of sediments deposited for urban development, the land begins to deform over time. According to Khan et al.^80^, a columnar portion of depth up to 152.4 m in Karachi's coastal region revealed subsurface rocks constituted of clay and silty sand layers. Thus, the burden of large metropolitan constructions, for instance, those in DHA-VIII, may place additional stress on the subsoil layers, resulting in increased deformation and compaction of the surface along the coast. Sector 7-A is more stable than the rest of the study area, with no subsidence. Sector 7-A is stable because there are no heavy construction activities. Sector 7-A has a displacement of 25.3 mm/year. While Sector 28 remained stable, some points were deformed. During the study period, sector 28 experienced the highest displacement of − 18.9 mm/year.

Due to large-scale water extraction, there was significant subsidence in Sherpao Colony, Radio Pakistan Colony, and the Milar area (Fig. 12). Sherpao Colony, Radio Pakistan Colony, and Milar areas have displacement rates of − 27.0 mm/year, − 31.7 mm/year, and − 30.9 mm/year, accordingly. One of the likely causes of subsidence in the study area is groundwater extraction for domestic purposes. The highest displacement in the Sherpao colony may result from industrial activities and heavy construction. These areas are home to the majority of the population. Fast population growth has been observed in these areas in recent years due to educational institutions such as military, medical, and engineering colleges.

### Groundwater extraction

The authors consider water extraction the primary cause of subsidence, as stated in^9,12,81–83^, with this phenomenon connected with soft clay soils in^9,82,83^. Seasonal impacts are identified in^83,84^, possibly related to hydrogeological factors and variations in groundwater level. Extraction of Groundwater for commercial and domestic purposes is one of the probable causes of subsidence in the study region^62,85^. Previous research has found that excessive groundwater causes ground liquefaction^27,46,86–91^. There is a water problem in Pakistan, and bore-wells and tube-wells are the primary sources of daily water consumption for people^92^. Water consumption has grown due to unplanned settlement expansion in major cities, and most households have built a bore-well to meet their demands. In addition, numerous enterprises operated by private and public entities, such as the agricultural and chemical sectors, are located in this city. These sectors, in instance, have significant groundwater consumption requirements.

**Figure 13:**
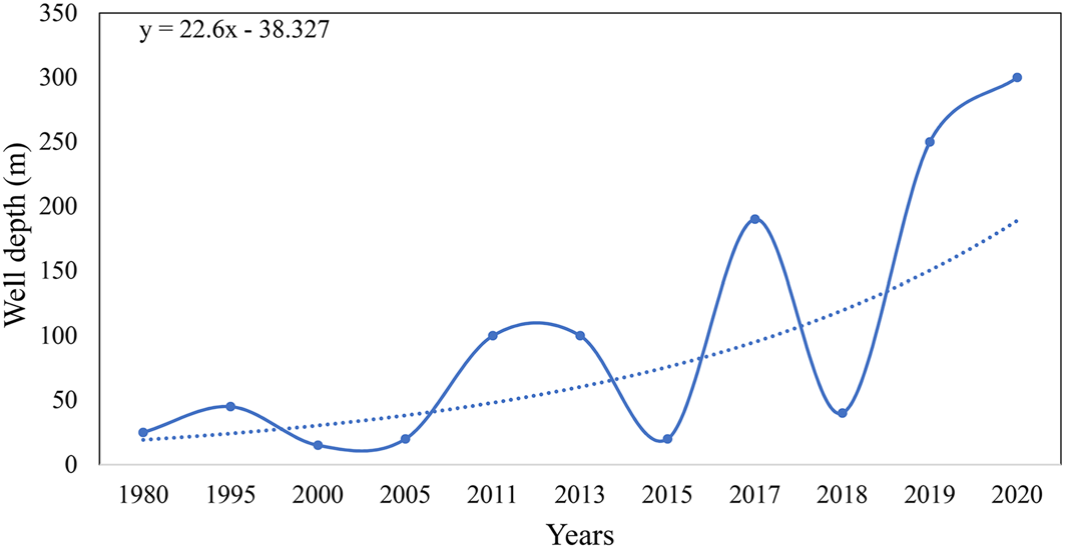
The trend of groundwater table in research area.

For the past three decades, Karachi has been plagued by a water supply and sanitation crisis. Dam and river water, which the Karachi Water and Supply Board regulate, are the primary water supply sources for both the industrial and domestic domains (KWSB). According to Khan et al.^80^, the gap between water demand and availability is growing by the day due to mismanagement, urban migration, and fast population growth in the research region (Tables 2, 3). As a result, individuals prefer to extract groundwater on a big scale. This is causing a drop in Karachi’s annual groundwater table, falling from 9 to 152 m^93^. Likewise, the level of water boreholes along the margins of the Malir River was around 6–8 m between 1960 and 1980, but it has since grown to 12–18 m^94^. It is concerning that, as time passes, every new well or bore must be drilled to a greater depth to extract groundwater because the water table has been declining at a pace of 22.6 m/year since 1980 (Fig. 13)^95^.

**Table 2:** Overview of water usage, demand, and availability in Karachi (Modified after Ashir and Khalid^84^; KWASB).

Parameters	Population
Total average available supply	550 MGD
Population^a^	16.05 million
Demand	1100 MGD
Water requirement per capita	54 gallons
Current shortfall (demand–supply)	650 MGD
The average duration of water supply available	2–4 h/day
Water currently available per capita	25 gallons

^a^Census, 2017, MGD: millions of gallons per day.

**Table 3:** Projections of future water demands based on KWSB study and Japan International Cooperation Agency (JICA 2008).

Parameters	2010	2015	2020	2025	Unit
Bulk water demand	741.1	903.8	1102.0	1300.3	MGD
Domestic consumption	272.6	362.3	497.3	655.3	MGD
Ratio of domestic consumption	60.4%	61.7%	63.2%	65.2%	%
Non domestic consumption	178.8	225.1	289.1	349.5	MGD
Population	18	22	27	32	x millions

**Figure 14:**
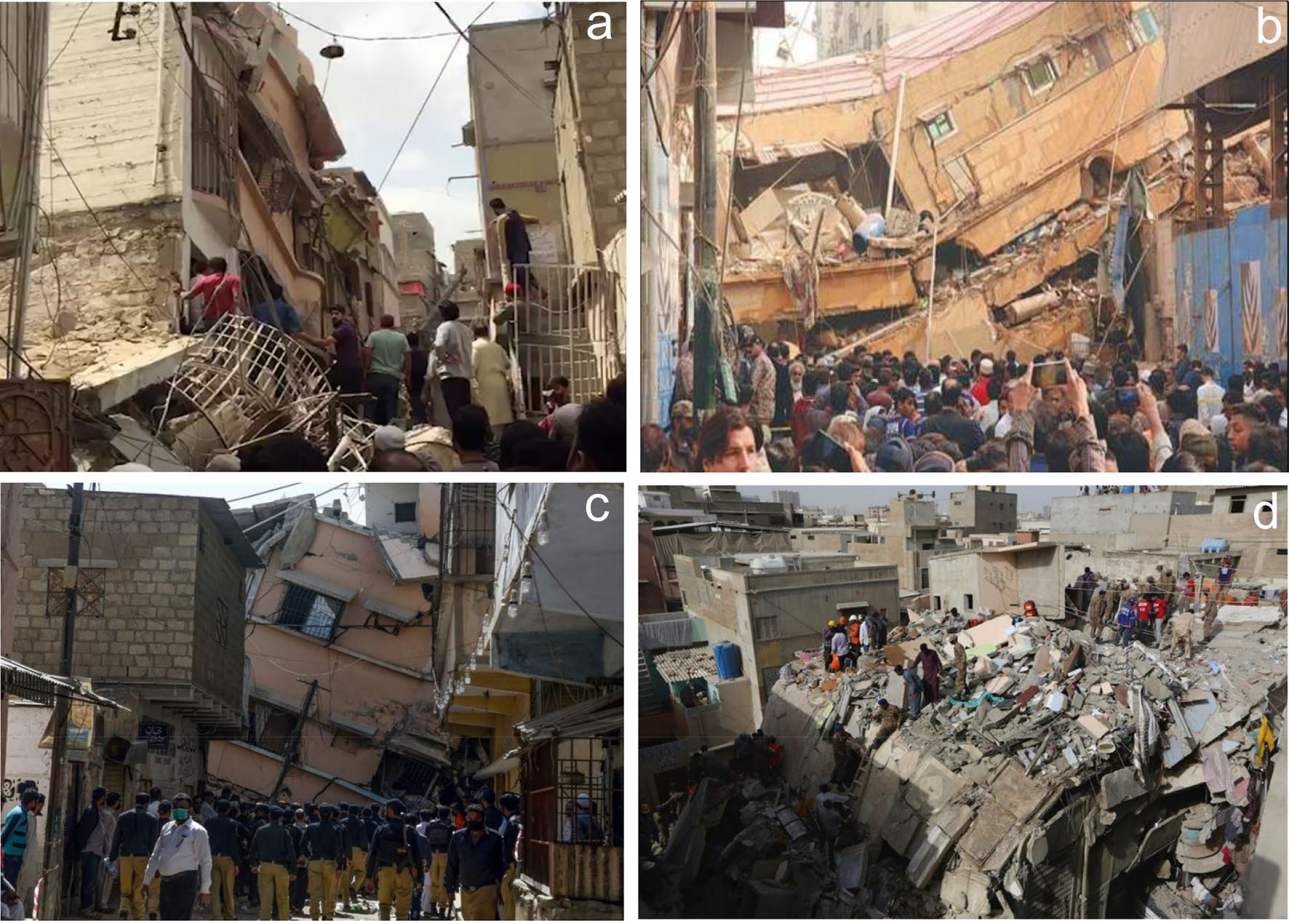
Land subsidence in different places of Karachi City. (**a**) Collapsed residential building at Rizvia Society, (**b**) Another collapsed at located in Soomro Street near Timber Market, Karachi City, (**c**) collapse residential building at Azizabad area, (**d**) Southern port city of Karachi.

Similar to prior research^80^, this was seen during field visits for this investigation. As a result, we might claim that large-scale groundwater abstraction in both industrial and residential zones has grown, which could be one of the causes of land deformation in Karachi. This has resulted in a few incidences of building collapse in the region (https://www.dawn.com/news/1539325), as illustrated in Fig. 14, where a whole street has begun to slump owing to groundwater abstraction in a residential district of Liaquatabad known as Azizabad (Fig. 14c).

The mathematical model can quantify land subsidence that occurred due to groundwater depletion. Bear et al.^96^ used the mathematical model for regional land subsidence due to pumping groundwater. Agarwal et al.^97^ depict the comparative study of groundwater-induced subsidence for London and Delhi Using PS-InSAR, which is based on the mathematical model. Khorrmi et al.^1^ used the Piezometric data and found the extreme subsidence in a populated city (Mashhad) detected by PS-InSAR considering groundwater withdrawal and geotechnical properties. Despite the lack of GPS-based in-situ data for land subsidence in the study area, field research and SAR-based assessment imply that land deformation in Karachi could be related to excessive groundwater abstraction combined with seawater intrusion. The findings of this study are consistent with other studies undertaken in various urban areas throughout the world to assess the association between groundwater and land subsidence^27,46,89–91,98^.

### Geological consideration

Soil consolidation qualities have been identified as a significant cause of subsidence worldwide^86^. It has been found that the majority of the Karachi metropolis has formed on alluvium deposits. The alluvium is generally made of sand, silty sand, sandy silt, deltaic, coastal, and eruptive mud deposits of recent age, with very little Clay^69^. The majority of the city, including institutions, the military school, and other residential and commercial structures, is constructed above alluvium deposits.

**Figure 15:**
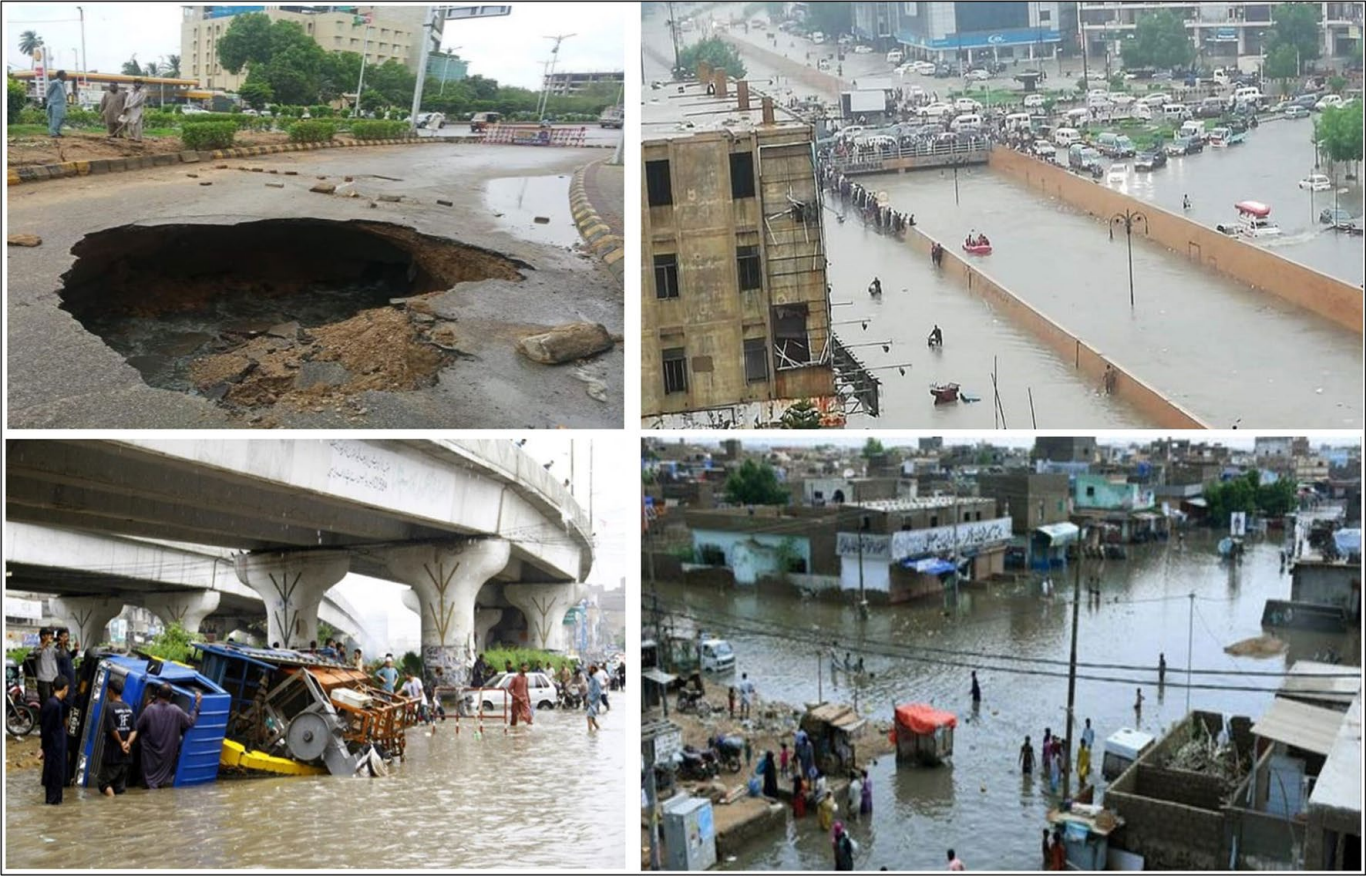
Water on the roads in Karachi city during raining.

There were no strong earthquakes documented in the research region throughout the inquiry period; however, movement along the Karachi arc’s active faults might cause surface deformation. However, it is crucial to remember that earthquakes have had epicenters between the Hyderabad highway and the river Malir^99^. According to the USGS Earthquake database, only four earthquakes were recorded between 1975 and 2021 within a 60-km radius of Karachi, with magnitudes ranging from 4 to 5 Mw. Furthermore, urban expansion and ongoing geologic activity (although in limited numbers) may exacerbate continuous land displacement in the region along the active Malir river fault and neighboring locations^100^. According to the findings, liquefaction happened primarily in quaternary alluvium layers (Fig. 15). It is probable that water precipitation into the subsurface, which permeates the subsurface layers and infrastructure stress, is to cause subsidence in the studied region.

Additionally, the connection between rainfall and liquefaction has been documented in^5,87^, and excessive precipitation disturbs the equilibrium of subsurface aquifers. This amount of precipitation, together with other causal variables, might impact subsidence. The high monsoon precipitation in the research area has previously been reported^58,62^. Subsurface aquifer refilling causes saturation of the subsurface layers, and a substantial association of subsidence in the study region with rainfall has previously been documented^58,62^.

**Figure 16:**
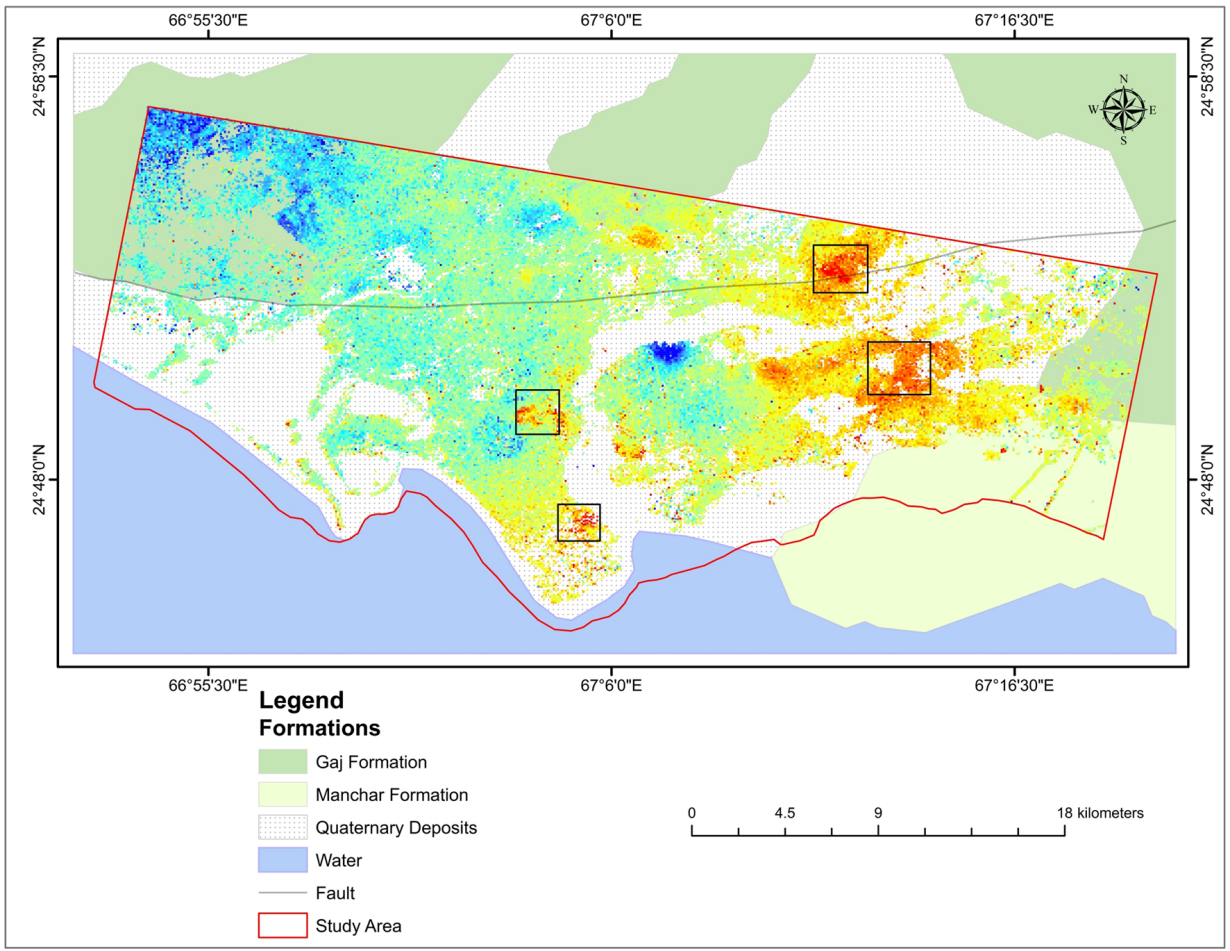
Subsidence on Geological map.

The above photographs (Fig. 15) were shot at various periods throughout the rainy season and showed flooding at several spots along the primary route. This route runs through the city’s heart; subsidence can be observed in Fig. 15 around the road (Fig. 15a). Figure 16 depicts the PS points overlay on a geological map of Karachi City. The black polygons, in this case, emphasize the subsidence on different parts of the research area, which is conspicuous in the Alluvium sediments. There were enough scatterers in the research region to estimate ground liquefaction. The red dots show subsidence in the research region in the polygon.

## Conclusions

PS-InSAR is a precise and accurate tool for monitoring urban structural collapse, ground subsidence, mining subsidence, landslides, and so on. However, specific noise effects and decorrelations can impact the final result. Meanwhile, our findings effectively indicated subsidence processes in the research region, although they may be improved by in situ data analysis and other approaches like Quasi-PS or SBAS. We observed ground subsidence in Karachi from November 2019 to December 2020 in this study, highlighting the capabilities of PS-InSAR to monitor time-series subsidence. The primary process factors have been thoroughly described, and different techniques to reduce noise and inaccuracies have been included throughout the processing. Various causal factors, such as subsurface geology, soil consolidation, groundwater extraction, and so on, have been studied. The study area’s subsidence maps suggest that Karachi is undergoing rising land subsidence. The data also show that subsidence is comparatively high in the city center, whereas it is significantly low in the eastern and western areas of the research region.

According to the findings of this study, the cumulative deformation in LOS ranged from − 68.91 to 76.06 mm/year. In contrast, the vertical deformation in LOS ranged from − 67.66 to 74.68 mm/year during the study period (2019–2020). The most obvious reasons for this appear to be fast population expansion in recent years, coupled with a rising need for daily-use water (industries and household) and groundwater extraction. Furthermore, subsurface geology with inadequate outflow, systems, and unauthorized building loads are significant reasons for ground subsidence in the studied region. Lastly, Geotechnical and well logs data are required to measure the more accurate subsidence rate in the future. It is also recommended that multi-scale research be done in the future to comprehensively examine ground subsidence and prevent significant harm in this area.

## Data Availability

Sentinel-1 data were obtained from https://asf.alaska.edu. The data presented in the study are available on request from the first author and corresponding.
